# Positive Selection in Bone Morphogenetic Protein 15 Targets a Natural Mutation Associated with Primary Ovarian Insufficiency in Human

**DOI:** 10.1371/journal.pone.0078199

**Published:** 2013-10-16

**Authors:** Sylvain Auclair, Raffaella Rossetti, Camille Meslin, Olivier Monestier, Elisa Di Pasquale, Géraldine Pascal, Luca Persani, Stéphane Fabre

**Affiliations:** 1 INRA, UMR85 Physiologie de la Reproduction et des Comportements, Nouzilly, France; 2 CNRS, UMR7247 Physiologie de la Reproduction et des Comportements, Nouzilly, France; 3 Université François Rabelais de Tours, Tours, France; 4 IFCE, Nouzilly, France; 5 Department of Clinical Sciences and Community Health, University of Milan, Milan, Italy; 6 INRA-ENVT, UMR 444, Laboratoire de Génétique Cellulaire, Castanet-Tolosan, France; 7 Institute of Genetic and Biomedical Research - UOS of Milan, National Research Council of Italy, Istituto Clinico Humanitas, Milan, Italy; 8 Laboratorio di Ricerche Endocrino-Metaboliche, Istituto Auxologico Italiano, Milan, Italy; Institut Jacques Monod, France

## Abstract

Bone Morphogenetic Protein 15 (BMP15) is a TGFβ-like oocyte-derived growth factor involved in ovarian folliculogenesis as a critical regulator of many granulosa cell processes. Alterations of the *BMP15* gene have been found associated with different ovarian phenotypic effects depending on the species, from sterility to increased prolificacy in sheep, slight subfertility in mouse or associated with primary ovarian insufficiency (POI) in women. To investigate the evolving role of BMP15, a phylogenetic analysis of this particular TGFβ family member was performed. A maximum likelihood phylogenetic tree of several TGFβ/BMP family members expressed by the ovary showed that BMP15 has a very strong divergence and a rapid evolution compared to others. Moreover, among 24 mammalian species, we detected signals of positive selection in the *hominidae* clade corresponding to F146, L189 and Y235 residues in human BMP15. The biological importance of these residues was tested functionally after site directed-mutagenesis in a COV434 cells luciferase assay. By replacing the positively selected amino acid either by alanine or the most represented residue in other studied species, only L189A, Y235A and Y235C mutants showed a significant increase of BMP15 signaling when compared to wild type. Additionally, the Y235C mutant was more potent than wild type in inhibiting progesterone secretion of ovine granulosa cells in primary culture. Interestingly, the Y235C mutation was previously identified in association with POI in women. In conclusion, this study evidences that the *BMP15* gene has evolved faster than other members of the TGFß family and was submitted to a positive selection pressure in the *hominidae* clade. Some residues under positive selection are of great importance for the normal function of the protein and thus for female fertility. Y235 represents a critical residue in the determination of BMP15 biological activity, thus indirectly confirming its role in the onset of POI in women.

## Introduction

BMP15 is an oocyte-derived growth factor belonging to the transforming growth factor-beta (TGFβ) superfamily, which is involved in ovarian follicular development as a critical regulator of many granulosa cell (GC) processes such as proliferation and steroidogenesis [1-4]. Similarly to other TGFβ/BMP factors, BMP15 is first synthesized as a pro-form processed by cleavage to liberate a mature form with biological activity and a large pro-domain [5,6]. After the removal of the signal peptide, the pro-protein dimerizes first and then undergoes proteolytic cleavage at a conserved RXXR cleavage site [2,3,7,8]. The pro-region is known to have an important role in the processing of the pro-protein by driving the dimerization and the subsequent secretion of the active mature dimers. BMP15 action in the ovary was first discovered in sheep with the evidence of six different natural “loss-of-function” mutations, two in the pro-region and four in the mature domain [4]. Heterozygous carrier ewes have increased ovulation rate, but homozygous carrier ewes show a blockade at the primary stage of folliculogenesis leading to sterility [9-12]. Unlike mutated BMP15 homozygous ewes, *bmp15* null female mice are fertile but exhibit a slight decrease in ovulation rate with minimal ovarian histopathological defects [13]. Interestingly heterozygous *bmp15* invalidated mice never exhibit increased ovulation rate as observed in sheep. Thus, the role of BMP15 appears to differ between species. This protein, associated with increased ovulation rate when altered in mono-ovulating sheep, seems to be dispensable in mouse, a poly-ovulating species. Recent findings are in agreement with this concept. Firstly, mouse seems to lack a biologically active Bmp15 molecule [14] and secondly, over-expression of a biologically active BMP15 in mice leads to accelerate folliculogenesis and causes an early decline in the ovarian reserve. Therefore, the lack of biologically active BMP15 during folliculogenesis in the wild type mice may be relevant to their polyovulatory nature [15]. Then, the *BMP15* gene has become a strong candidate gene for genetic alterations associated with human ovarian pathologies. In women, mutations in BMP15 have been found associated with both primary and secondary amenorrhea in several primary ovarian insufficiency (POI) cohorts with prevalence between 1.5% and 15% [16]. POI, a more appropriate definition of premature ovarian failure, is a disorder associated with female infertility affecting 1 to 2% of women under 40 years old, thus representing one major cause of female infertility [17]. Up to now, more than a dozen of mutations in BMP15 have been described associated with POI [16]. Most of the POI-associated BMP15 mutations are missense mutations found at the heterozygous state and located in the pro-region part of the protein. When tested functionally, these mutations hampered the protein processing and the biological activity, consistent with a mechanism of haploinsufficiency [18]. 

Regarding all the above data, the *BMP15* gene is the target of numerous mutations associated with altered ovarian function in different species, which highlights its crucial role in female fertility but with species-specific differences. To our knowledge, experimental and functional results of positive selection of gene involved in vertebrate reproductive functions are missing. The central hypothesis being examined in this paper is to determine if BMP15 has evolved under positive selection, and if this evolution had consequences on the control of ovarian function in mammals. 

## Results and Discussion

### BMP15 evolution among TGFß family members

**Figure 1:**
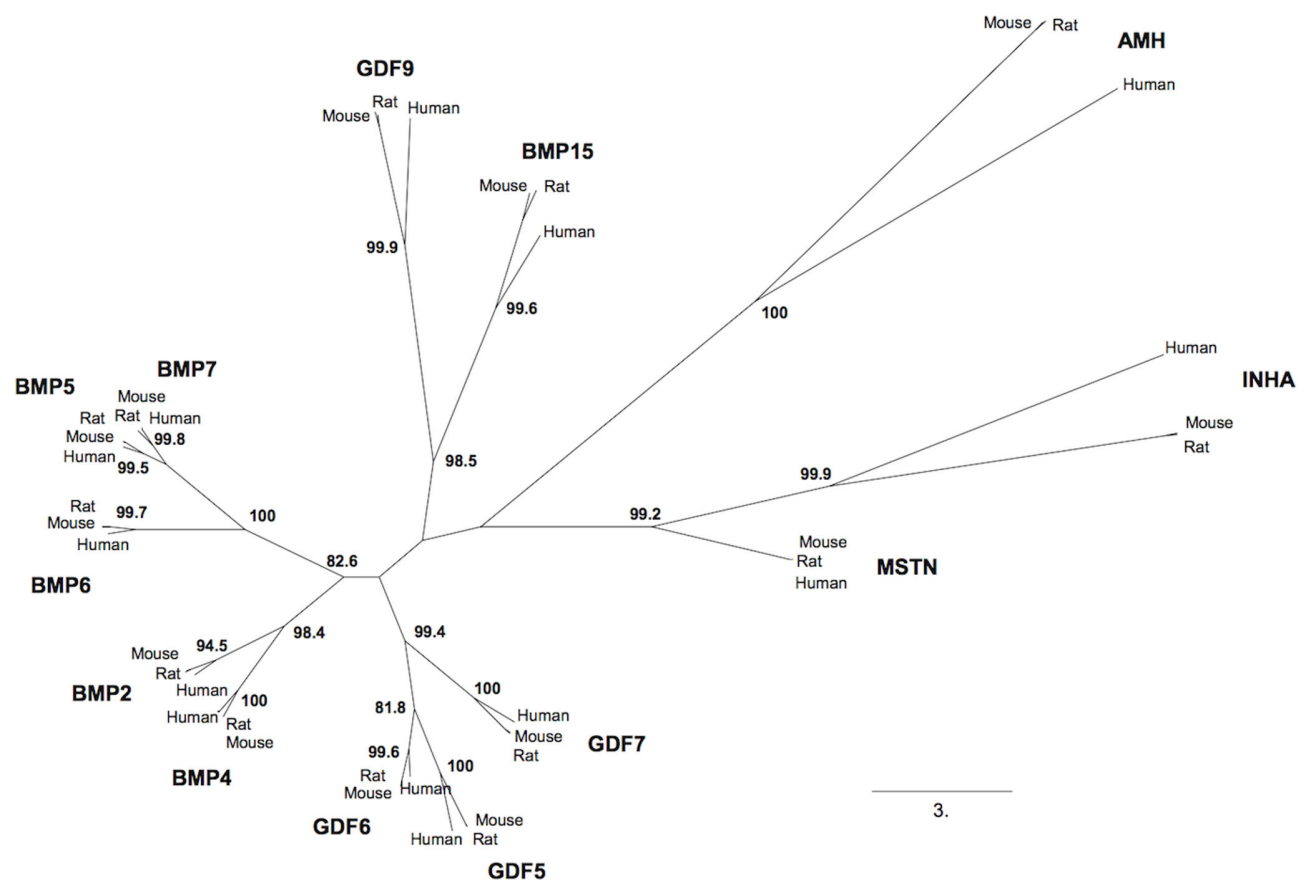
BMP15 evolution among TGFß/BMP family members. Phylogenetic tree of thirteen members of the TGFβ/BMP superfamily expressed by the mammalian ovary reconstructed using maximum likelihood. Bootstrap values are given when nodes are strongly supported (>80%). The scale represents the substitution rate.

We performed a phylogenetic analysis of sereval TGFβ/BMP family members expressed within the ovary throughout selected species: human, mouse and rat (Figure 1 and Table S1). The maximum likelihood (ML) tree we obtained is consistent with previous TGFß phylogenies made with the entire pre-pro-proteins sequences [19,20]. BMP15, as well as GDF9, INHA and AMH (all marker genes of the ovarian function), evolve more rapidly and are more divergent between species than the other members of the TGFβ superfamily as indicated by their high length branches. Specifically, BMP15 evidenced a very strong divergence and a rapid evolution compared to other BMP genes expressed in the ovary like other genes encoding proteins involved in reproductive functions as previously shown [21,22]. 

### BMP15 positive selection events

**Figure 2:**
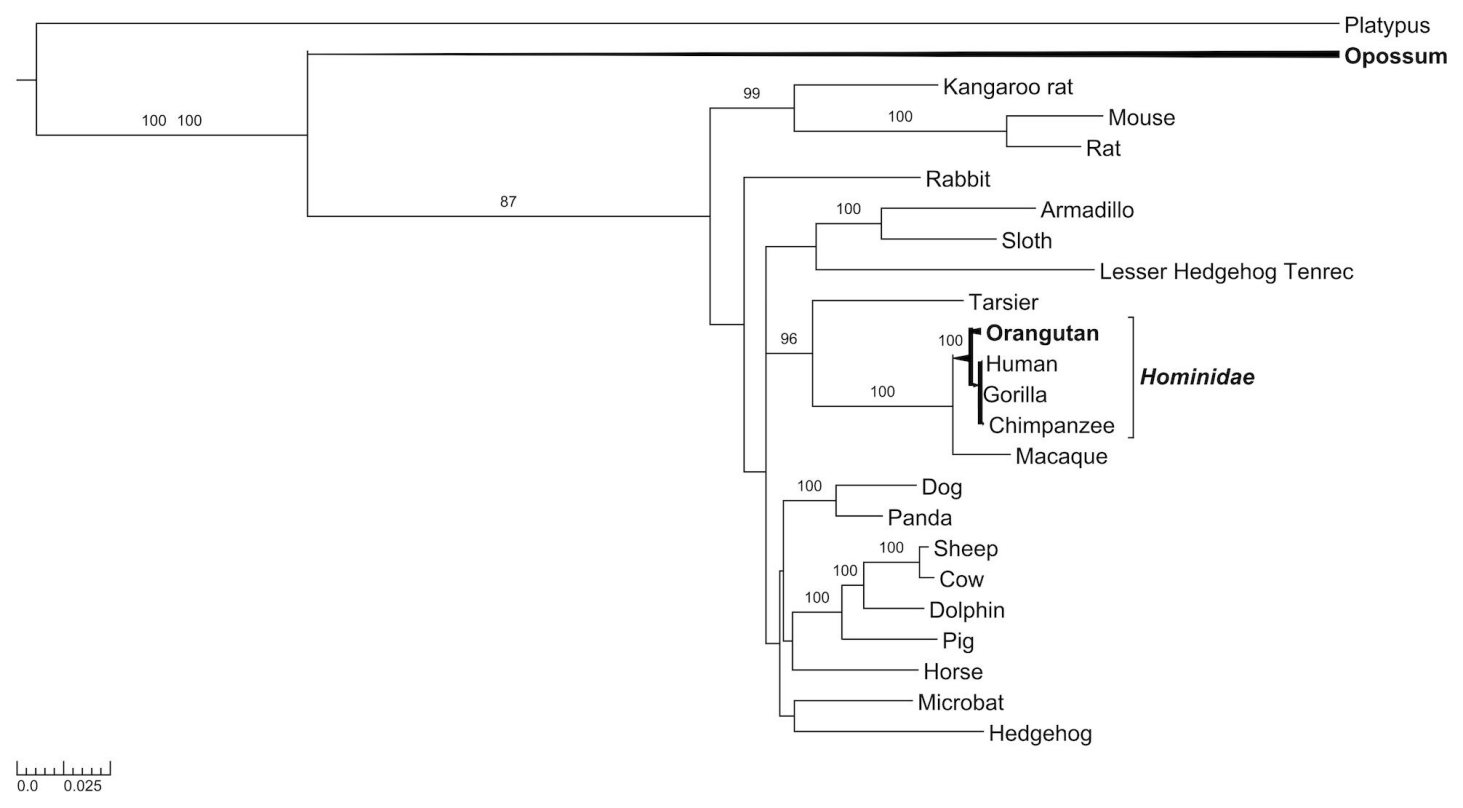
BMP15 positive selection pressure among mammals. Phylogenetic tree of BMP15 from 24 mammals species reconstructed using TreeBeST and rooted by minimizing with the number of duplications and losses. Bootstrap values are given when nodes are strongly supported (>80%). Branches or species that have a significant LRT for positive selection calculation are indicated in bold (threshold of *q*= 5% of false positives). The scale represents the substitution rate.

**Figure 3:**
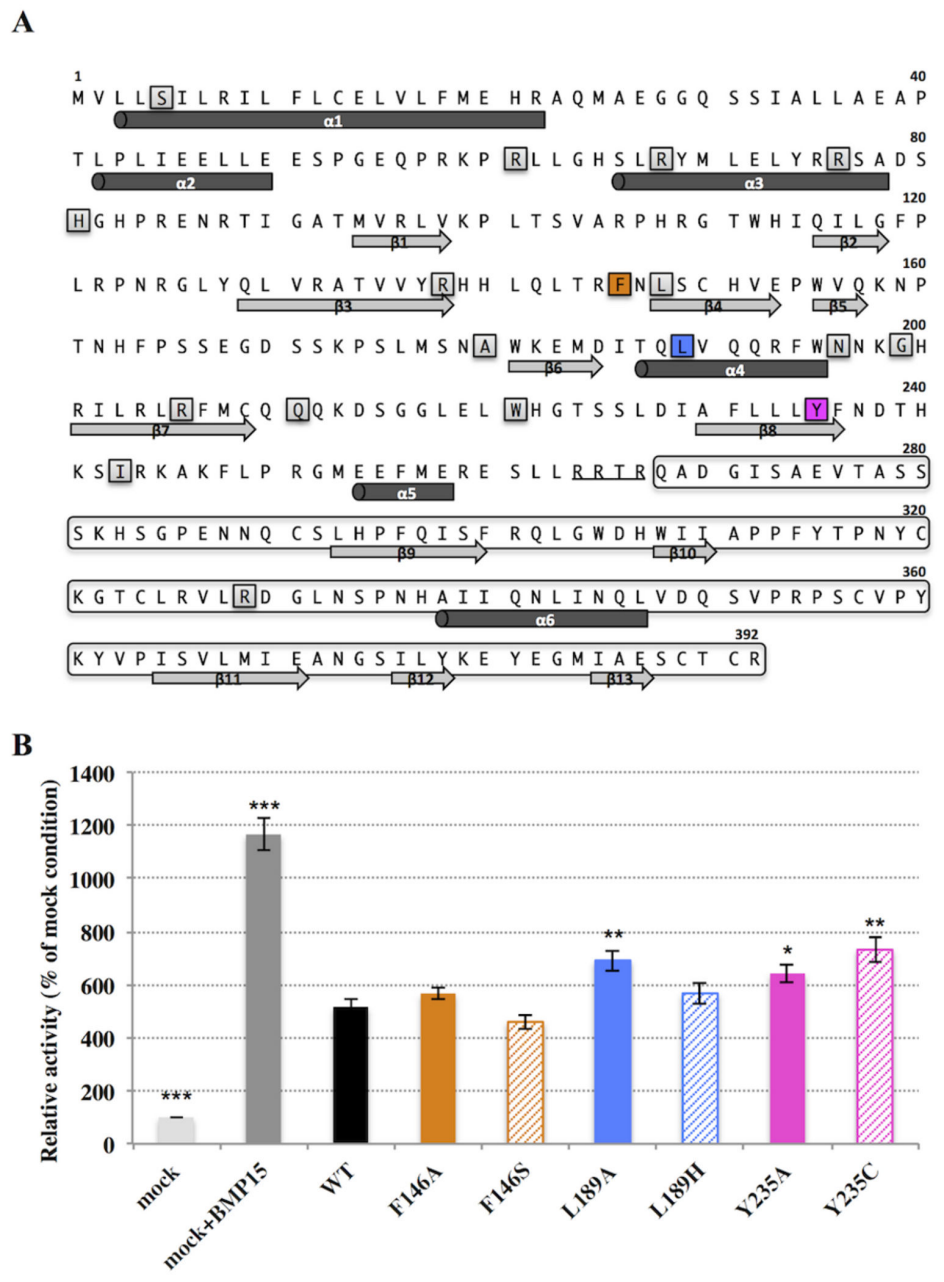
Functional analysis of amino acids under positive selection in human BMP15. A, Human BMP15 full sequence showing prodomain, cleavage site (underlined) and mature peptide (framed sequence); known human variants for BMP15 found in POI patients (framed grey highligthed); and identified amino acids under positive selection (framed colored highlighted): BMP15^F146^, BMP15^L189^, BMP15^Y235^. B, *In* *vitro* reporter luciferase assay from COV434 granulosa cells transiently transfected with empty vector +/- 100ng recombinant human BMP15 (mock +/- BMP15) or wild type human BMP15 expressing vector (WT) or the different BMP15 variants vectors obtained by directed-mutagenesis of residues under positive selection. Results are expressed as the mean (±SD) of 4 independent experiments. Differences between means were analyzed by one-way ANOVA by comparing each condition to WT (*, p<0.05; **, p<0;01; ***, p<0.001).

Given that positive selection is often associated with branch length and fast gene evolution [23], and that genes involved in reproduction evolve faster and often evidenced positive selection [24-26], we carried out positive selection analyses on BMP15. We used the BMP15 sequences from 24 mammals (Figure 2 and Table S2) analyzed by branch-site models from PAML packages [27] within the PhyleasProg web server [28], in order to determine whether the different species of the phylogenetic tree underwent selective pressures and to detect signals of local episodic positive selection. Those analyses were applied to the full sequence, the pro-region and the mature form of BMP15. Results for the mature form did not show any sites under positive selection; this result is not surprising since this domain is highly conserved among species. We tested all branches of the phylogenetic tree of BMP15 pro-region and three branches appeared to be significant: opossum, orangutan and *hominidae* (Figure 2 and Table S3). We identified five positively selected sites for opossum (W55, S73, D89, G165 and P187); two of these five amino acids (S73 and P187) were also identified by the calculation of positive selection using the full sequence of BMP15. We identified three positively selected sites for the orangutan (I25, R146 and R215) and three for the *hominidae* clade, corresponding in human sequence to F146, L189 and Y235 (Figure 3A). It has been reported that GC biased gene conversion (gBGC) [29] could increase the *dN*/*d*_*S*_ *ratio*, especially in primates and cause false positive detection of amino acid in branch-site positive selection study [30,31]. We know that in *hominidae* the *BMP15* third-codon position GC rate (53%) is high compared to the gene average in this clade (46%) [32]. However, a recent study by Gharid and Robinson-Rechavi [33] shows that the GC content of a sequence did not significantly affect the branch site model.

### Biological activity of the BMP15 positively selected sites

**Figure 4:**
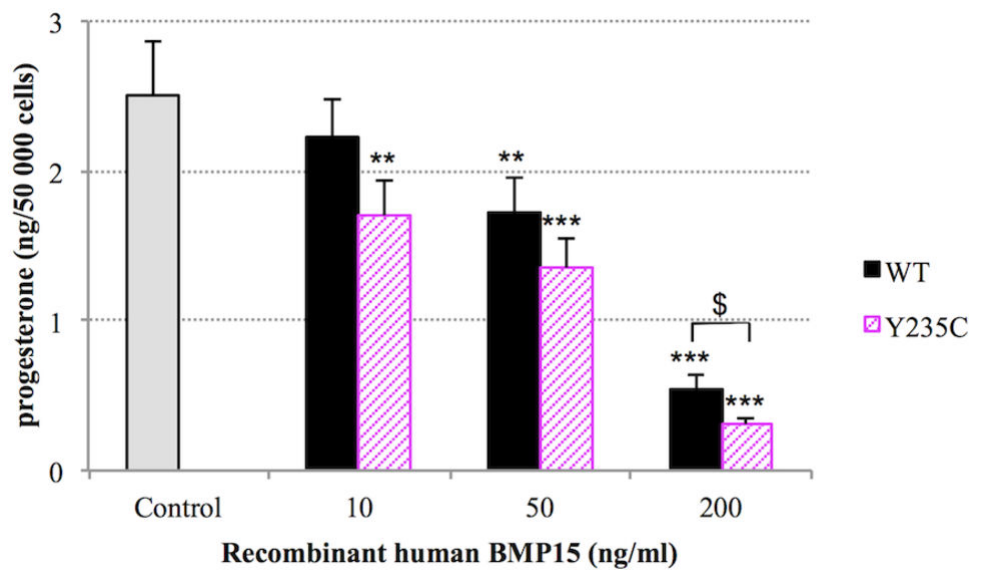
Recombinant BMP15 inhibition of progesterone secretion. Granulosa cells from small antral (1–3 mm in diameter) ovine follicles were cultured for 96 h in serum-free conditions. Cultures were performed in the absence (control) or in the presence of increasing dose (10, 50, 200ng/ml) of wild type recombinant human BMP15^wt^ or BMP15^Y235C^ mutant. Each treatment was tested in triplicate in each of 5 independent experiments. The results represent the amount of progesterone secreted by 50 000 cells between 48 h and 96 h of culture (mean ±SEM). Dose effect was analyzed by one-way ANOVA by comparing each dose to control (**, p<0.01; ***, p<0.001). Mutation effect was analyzed by Student t-test within each dose ($, p<0.05).

In order to check the functional role of these three amino acids in the human BMP15 protein, we performed a luciferase reporter assay in the granulosa-derived COV434 cell line, as previously described [18]. We analyzed biological activities of the human wild type BMP15 compared to those of mutant variants that have been obtained by site-directed mutagenesis for the three positions under positive selection. One set of mutants was obtained by the systematic substitution of the human wild type amino acid under positive selection by an Alanine (F146A, L189A and Y235A). The second set of mutants was obtained by replacing the human wild type amino acid by the one that is the most represented at the same position in the other species within the BMP15 multiple alignment used for phylogenetic analysis (F146S, L189H and Y235C, Figure S1). As positive controls, COV434 cells expressing the luciferase reporter gene showed an activation of the BMP signaling pathway when stimulated with 100 ng of the exogenous recombinant human BMP15, or when transiently transfected with the wild type human BMP15 expressing construct (Figure 3B). Among our positively selected variants, BMP15 activity was not affected by the F146 residue mutations. In contrast, the transfection of Y235A and Y235C mutants showed a significant enhancement of the BMP signaling pathway of 24 to 42% compared to the wild type. Moreover, L189A also increased the BMP15 activity by 34% (Figure 3B). Accordingly, the human BMP15 secondary structure prediction by PsiPred [34] revealed that the F146 site is situated in a coil region, while L189 and Y235 are situated in the fourth alpha helix and in the eighth beta sheet, respectively (Figure 3A). This was confirmed by the crystallographic structure of TGFß1[35] and, thus these positions are suspected to affect the three-dimensional structure of BMP15. Additionally, purified recombinant human BMP15 proteins were assayed on their ability to modulate the progesterone synthesis by granulosa cells. When compared to wild type, recombinant human BMP15^Y235C^ was dose-dependently more potent in inhibiting progesterone secretion by ovine granulosa cells in primary culture (Figure 4). Indeed, the significant biological action of BMP15^Y235C^ started at 10ng/ml compared to 50ng/ml for the wild type protein. Moreover, at 200ng/ml the inhibiting action of BMP15^Y235C^ was significantly higher than wild type. These results were in agreement with the increased signaling activity of the BMP15^Y235C^ mutant in the COV434 cell assay. 

Overall, these findings are consistent with the idea that some residues under positive selection are of key importance in the function of the protein. Particularly, Y235 represents a critical residue in the determination of BMP15 biological activity and BMP15^Y235C^ was the first mutation found associated with POI in human [36]. Interestingly, the present functional analysis of this position indicates that its alteration enhanced the BMP15-dependent transcriptional activity associated with enhanced action on granulosa cell steroidogenesis. In contrast, BMP15^Y235C^ was unable to increase granulosa cells proliferation as previously shown [36]. This could indicate a discriminant action of this mutation on BMP15-dependent signaling on proliferative vs. differentiative function of granulosa cells. The Y235 residue is situated in the pro-region of BMP15, and it is known that the pro-region is able to modulate the expression and activity of the mature protein involving posttranslational modifications. Chimeric constructs with human BMP15 pro-region and mouse mature region are able to produce mouse BMP15 protein in a better way compared to the native mouse BMP15 pre-protein [14]. Moreover, the substitution of specific residues in the pro-domain of murine and ovine BMP15 protein lead to a decrease of the expression and activity of the chimeric protein compared to the wild type form, when tested on COV 434 cells [37]. Based on these observations, the Y235C mutation is probably affecting BMP15 activity through the posttranslational processing modification of the protein previously shown [36].

When tested functionally, most of the other BMP15 mutations associated with primary or secondary amenorrhea in POI women (Figure 3) hampered the protein processing and the biological activity through the same COV434 luciferase test [18]. This indicates that the mechanism behind these POI cases is associated with a loss of BMP15 function. Nevertheless, the present results may support an inverse hypothesis i.e. an enhanced BMP15 activity could also be related to POI. In most of the cases, ovarian insufficiency occurs because of an anticipated depletion of the primordial follicular pool within the ovaries, due to a diminished size of the pool, an accelerated atresia, or an altered recruitment. This fits very well with the phenotype observed in mice over expressing a functional BMP15 [15]. Intriguingly, C235 is the wild type residue observed in chimpanzee (*Pan troglodytes*). One could hypothesize that the Y235 to C235 substitution could be responsible of the high rate of dizygotic twin observed in this species. Indeed, the chimpanzee dizygotic twinning rate (2.46%) is over twice the human average. This amino acid substitution may be positively selected due to a high selection pressure caused by the *P. troglodytes* heteropaternity sexual behavior [38]. 

Taken together, our data suggest that BMP15 amino-acids under positive selection (L189 and Y235) may have a repressive effect on BMP15 biological activity and then may contribute to limit the anticipated depletion of primordial follicular pool observed in POI patients. Usually, biological activities of positive selective sites are associated with an increase of biological activity. As example, the deletion of the positively selected amino acids in the SPRY domain in TRIM5α that has protective effects against viral infections in primates, leads to the loss of its antiviral activity against HIV-1 and SIV viruses [39]. Thus, to our knowledge, our study on BMP15 is the first example of amino acids under positive selection with a repressive effect. 

In this study, we determined that oocyte-expressed *BMP15* gene has evolved faster than other members of the TGFß family and was submitted to a positive selection pressure in the *hominidae* clade. Some residues under this positive selection are of great importance for the normal function of the protein and thus for female fertility. Y235 represents a critical residue in the determination of BMP15 biological activity, confirming its role in the genetic determinism of POI in women through a complex and still undefined derangement of BMP15 paracrine function.

## Materials and Methods

### Phylogenetic and evolutionary analyses

Thirty-nine protein sequences belonging to the TGFβ superfamily were retrieved from Ensembl for human, mouse and rat (Table S1). Multiple sequence alignments were performed using the MUSCLE algorithm [40]. Phylogenetic tree was reconstructed using maximum likelihood (ML) in PhyML 3.0 under the LG model [41]. Bootstrap values [42] were estimated with 1000 replications. BMP15 coding and protein sequences were retrieved from Ensembl database Release 70 ({http://www.ensembl.org/index.html) from 23 mammal species: armadillo (*Dasypus novemcinctus*), chimpanzee (*Pan troglodytes*), dog (*Canis familiaris*), dolphin (*Tursiops truncatus*), gorilla (*Gorilla gorilla*), hedgehog (*Erinaceus europaeus*), horse (*Equus caballus*), human (*Homo sapiens*), kangaroo rat (*Dipodomys ordii*), lesser hedgehog tenrec (*Echinops telfairi*), macaque (*Macaca mulatta*), microbat (*Myotis lucifugus*), mouse (*Mus musculus*), opossum (*Monodelphis domestica*), orangutan (*Pongo abelii*), panda (*Ailuropoda melanoleuca*), pig (*Sus scrofa*), platypus (*Ornithorhynchus anatinus*), rabbit (*Oryctolagus cuniculus*), rat (*Rattus norvegicus*), sloth (*Choloepus hoffmanni*), tarsier (*Tarsius syrichta*), cow (*Bos taurus*). An additional sequence was retrieved from NCBI database (http://www.ncbi.nlm.nih.gov/) for one mammal: sheep (*Ovis aries*) (Table S2). Phylogenetic tree and evolutionary analyses were computed on BMP15 pro-regions (i.e. from the N-terminus to the consensus cleavage site -RXXR-), on mature domain (i.e. from the consensus cleavage site –RXXR- to the C-terminus) and on full coding sequence. Multiple sequence alignment (MSA), tree reconstruction as well as positive selection calculation are then computed using the PhyleasProg web server v3.0 with the “fine” and “orthologs only” options [28]. The PhyleasProg web server uses the codeml program from the PAML package [27] to evaluate the ratio ω (ratio of non-synonymous/synonymous substitution rates, *dN*/*d*_*S*_), which is a measure of selective pressure. Values of ω<1, =1 and >1 are indicators of purifying selection, neutral evolution and positive selection, respectively. We used branch-site models [43,44], which permit different branches (or sets of branches) to evolve under different selective constraints. All branches of the phylogenetic tree are tested as foreground branch. Two models are used, one called “alternative” in which the foreground branch may have some sites under positive selection, and one called ‘null’ in which positive selection is not allowed for the foreground branch. For the “alternative” model, three classes were defined: *ω*_0_: dN/dS<1, *ω*_1_: dN/dS=1 and *ω*_2_: dN/dS≥1, while in the “null” model, *ω*_2_ was fixed to 1. Calculations are performed on a 272 codons length in order to keep the most possible informative sites (Figure S1). Likelihood ratio tests were used to compare log likelihood values between the two models. In the context of multiple testing, we calculated *q-value* measures as an extension of the false discovery rate, using the *q-value* package of R [45]. The *q-value* attached to each individual branch described the expected proportion of false positives among all branches equal to or more extreme than the observed one. Therefore, the thresholding of the estimated *q-values* at alpha level =10% produced a list of significant branches so that the expected proportion of false positives was alpha. Bayes Empirical Bayes (BEB) method [44] was used to estimate posterior probabilities of selection on each codon, probabilities >0.90 were considered for further study.

### Construction of BMP15 expression plasmids

BMP15 gene variants were introduced by site-directed mutagenesis into the pCSBMP15wt vector, containing a full-length human BMP15 wild type cDNA [18]. Mutagenesis reaction for each variant was performed using the QuickChange Site-Directed Mutagenesis kit (Stratagene) and specific couples of primers (Table S4). Two series of mutagenesis were performed, the first one by changing the amino acid under positive selection by an Alanine (chemically inert) and the second one by the most frequently observed amino acid in protein alignment of BMP15 among all studied species (Figure S1). All mutations were checked by DNA resequencing.

### Transient transfections and luciferase reporter assay

A COV434 human granulosa cells line [46] stably expressing the BMP responsive element (BRE) - luciferase reporter was seeded at a density of 500000 cells/well in 12-well plates in DMEM supplemented with 10% FCS. When subconfluent, cells were transfected in triplicate with the wild type or mutant pCS2 expression vector (500 ng/well) by using Fugene HD (Roche Applied Sciences, Indianapolis, IN) following manufacturer’s protocol. The pCS2 empty vector was used as negative control. Approximately 24 hr after transfection, the medium was replaced with 1% serum medium with 100 ng/ml of rhBMP-15 (R&D Systems, Minneapolis, MN) added only in positive control wells. After 16 hr of treatment, cells were rinsed with ice-cold PBS and then lysed with 200 ml of Passive Lysis Buffer 1X (Promega). Cell lysates from each well were centrifuged at 12,000 rpm for 2 minutes to pellet the cell debris and 20 μl of the supernatants were then assayed for luciferase activity using the Dual Luciferase reporter Assay kit (Promega). Luminescence in relative light units (RLU) was measured for 10 s in a Fluoroskan Ascent instrument (Labsystems, Oy, Finland). Results and statistical analysis (one-way ANOVA followed by Dunnett’s post-hoc test, GraphPad Prism v6.0) were then calculated as the mean (±SD) relative to pCS2-BMP15 wild type expression vector in triplicate in 4 separate experiments. Differences with P > 0.05 were considered as not significant.

### Ovine granulosa cells culture and progesterone assay

Five cyclic Romanov ewes were treated with intravaginal progestagen sponges (fluorogestone acetate, 40 mg, Intervet, Angers, France) for 13 days in order to synchronize estrus. The ovaries were recovered at slaughtering in the late follicular phase, 36 h after sponge removal. This procedure was approved by the ‘‘Direction Départementale des Services Vétérinaires d’Indre-et-Loire’’ (approval number C37-175-2) for the agricultural and scientific research agencies INRA (French National Institute for Agricultural Research) and CNRS (French National Center for Scientific Research), and conducted in accordance with the Guide for the Care and Use of Agricultural Animals in Research and Teaching. Primary granulosa cells were recovered from ovarian follicles of 1-3mm in diameter and seeded at 100 000 viable cells/well in 96-well plates and cultured for 96 h at 37 °C with 5% CO2 in serum- free McCoy 5a medium (Sigma, L’Isle d’Abeau Chesnes, France) as previouly described [47]. Cultures were performed with or without 10, 50 and 200ng/ml of recombinant human BMP15 or BMP15^Y235C^ prepared previously [36]. Culture media were partially replaced (180 over 250 μl) at 48 h. Media conditioned between 48 and 96h of culture were collected at 96h and stored at -20 °C prior to the progesterone assay. At the end of the culture period, the number of cells per well was estimated after trypsinization with a hemacytometer under a phase contrast microscope. Progesterone amounts in the culture media from each experiment were measured by radioimmunoassay in the same assay, as described [48]. The limit of detection of the assay was 12 pg/tube and the intra-assay coefficient of variation was 10%. The results are expressed as the amount of progesterone secreted per 50 000 cells recovered at the end of the culture period. Results were calculated as the mean (±SEM) of triplicate in 5 separate cultures. Statistical analysis (GraphPad Prism v6.0) for BMP15 dose-effect was performed by one-way ANOVA. Statistical significance was given relatively to the control condition after Dunnett’s multiple comparison tests. Mutation effect was tested by Student t-test within each BMP15 dose. Differences with P > 0.05 were considered as not significant.

## Supporting Information

Figure S1 **Multiple alignment of BMP15 pro-regions in 24 mammals using the MUSCLE algorithm.**
(PDF)

Table S1 **Ensembl protein identification numbers of the 39 members of the TGFbeta family used to draw the phylogenetic tree.**
(PDF)

Table S2 **Ensembl identification numbers (gene, transcript and protein) of the 24 mammalian orthologs of BMP15 used in the branch-site model for positive selection determination.**
(PDF)

Table S3 **Branch-site model parameters for positive selection determination.**
(PDF)

Table S4 **Sence and antisense primers used fot human *BMP15* mutagenesis.**
(PDF)
