# Supplementary material for: Positive Selection in Bone Morphogenetic Protein 15 Targets a Natural Mutation Associated with Primary Ovarian Insufficiency in Human
Source: PLoS One. 2013 Oct 16;8(10):e78199. doi: 10.1371/journal.pone.0078199 (PMC3797742; doi:10.1371/journal.pone.0078199)
Supplement: Figure S1 — Multiple alignment of BMP15 pro-regions in 24 mammals using the MUSCLE algorithm. (PDF) [file pone.0078199.s001.pdf]

Figure S1: Multiple alignment of BMP15 pro-regions in 24 mammals using the MUSCLE algorithm.

```
                                  10         20         30         40         50
                        ....|....| ....|....| ....|....| ....|....| ....|....|
Human                   MVLLSIL-RI LFLCELVLFM EHRAQMAEGG QSSIALLAEA PTLPLIEELL
Chimpanzee              MGLLSIL-RI LFLCELVLFM EHRAQMAEGG QSSIALLAEA PTLPLIEELL
Gorilla                 MVLLSIL-RI LFLCELVLFM EHRAQMAEGG QSSIALLAEA PTLPLIEELL
Orangutan               MVLLSIL-RI LFLCELVLFM EHRAQIAEGG QSSIALLAEA PTLPLIEELL
Macaque                 MVLRSIL-RI LFLCELVLFM EHRAQMAEGR QSSIALLAEA PTLPLIEELL
Mouse                   MALLTIL-R- ILLWGVVLFM EQRVQMAKPG WPSTALLADD PTLPSILDLA
Rat                     MALLTIL-R- ILLWGMVLFM EHKVQMAKVE WPSTTLLAEN PTLPSSLDLA
Kangaroo rat            MALLTIL-R- ILFCGLVFLM EHRVQMVKAE QPSVAPLAED PALPLIQELL
Rabbit                  MALLSIL-RA LLLWGLVIFM EHRVQAAKVG QSSIALLAEA PTLPLIRELL
Sheep                   MVLLSIL-RI LL-WGLVLFM EHRVQMTQVG QPSIAHLPEA PTLPLIQELL
Cow                     MVLLSIL-RI LLLWGLVLFM EHRVQMTQVG QPSIAHLPEA PTLPLIQELL
Pig                     MVLLSII-RT LLLWGLVLFM EHRVQMTQVG QPSVALLPEA CTLPLIRELL
Panda                   MVLLSIL-RI LL-WGLVLFM EHKVQMAKVG QSSNALMADA PSLPLIRELL
Dog                     MVLLSIL-RI LL-WGLALFM EYRVQMAKVG QPSNALMADT PSLPLIRELL
Horse                   MVLLSIL-RI LL-WGLVLFR EHRVQMAKVG QPSIALPAEV PTLPLILELL
Dolphin                 MVLLSIL-RI LLLWGLVFFM EHVVQMTQVG QPSVALLPEA PTLPLIRELL
Armadillo               MLLFGIL-RV LLVSGLVIFV EHRVQMARVR ESSIALLAET PTLPLIRELL
Lesser hedgehog tenrec  MVLLSIL-KT ILLWKLIIFM EHRVQMARVG QP---LLAEG PALPLIQELL
Hedgehog                MVLVSIL-RF LLFWGLVLFM EHGVQMAKAG KPSLALLAEA PTLPLIWELL
Tarsier                 MVLFSIL-RI LLLWKLVLII EHGAQMAKAG QPSIALLTEA PTLPLIRELL
Microbat                MVLLSSF-RI LLLWGLVLFT EHRVQMGTVG KHSTGPLAEA PTLPLIQELL
Sloth                   MVLFSIL-RI LLVWGLMIFV EHKVQMARVR QSSIALPAKA STLPLIQELL
Platypus                MALLRFLFLA LLPWELAF-- ---RGAGMNP QS----LAAA SELSFLQELL
Opossum                 ----SME-AD WLLWALLFVL ELSSLKGTNS QDAAAPAHVA LQLPLLRALL

                                  60         70         80         90        100
                        ....|....| ....|....| ....|....| ....|....| ....|....|
Human                   EESPGEQPRK PRLLGHSLRY MLELYRRSAD SHGHPRENRT IGATMVRLVK
Chimpanzee              EESPGEQPRK PRLLGHSLRY MLELYRRSAD SHGHPRENRT IGATMVRLVK
Gorilla                 EESPGEQPRK PRLLGHSLRY MLELYRRSAD SHGHPRENRT IGATMVRLVK
Orangutan               EESPGEQPRK PRLLGHSLRY MLELYRRSAD SHGHPRENRT IGATMVRLVK
Macaque                 EESPDEQPTK PRLLGHSLQY MLELYRRSAD SHGHPRENRT IGATMVRLVK
Mouse                   KEAPGKE-MK QWPQGYPLRY MLKLYHRSAD PHGHPRENRT IGAKMVRLVK
Rat                     KEAPGKE-MK QWPQGYPLRY MLKLYQRSAD PHGHPRENRT IGAKMVRLIK
Kangaroo rat            EDAPRKQQIK QWPQGYPLRY MLKLYQRSAD PHGHPRENRT IGATMVRLVR
Rabbit                  EEAPDKQQRK PQLQGHPLRY MLELYQRSAD PHGHPRENRT IGATMVRLVR
Sheep                   EEAPGKQQRK PRVLGHPLRY MLELYQRSAD ASGHPRENRT IGATMVRLVR
Cow                     EEAPGKQQRK PRILGHPLRY MLELYQRSAD ASGHPRENRT IGATMVRLVR
Pig                     EEAPGKQQRK PQVLGHPLRY MLELYQRSAD ARGHPRENRT IGATMVRLVR
Panda                   EEAPGKQQRK SQVLGHPLRY MLELYQRSAD AHGHPRENRT IGATMVRLVR
Dog                     EEAPGKQQRK PQVLGHPLRY MLELYQRSAD ARGHPRENRT IGATMVRLVR
Horse                   EEAPAKQQGK PQVLGHPLRY MLELYQRSAD AHGHPRENRT IGATMVRLVK
Dolphin                 EEAPGKQQRK PRVLGPPLRY MLELYQHSAD ESGHPRENRT IGATMVRLVR
Armadillo               EEAPGKQQRK PWLLGHPLRY MLELYQRSAD LHGHPRENRT LGATMVRLVR
Lesser hedgehog tenrec  EEAPSRQQRK PRFLGHSLQY MLELYQRSAD PWGHPRKNRT IGATMVKLVR
Hedgehog                EEAPVKQQRK PHVLGHPLRY MLELYQRSAD THGHPRENRT IGATMVRLVR
Tarsier                 EEAPKQE--K PQLLGHPLRY MLELSQRSAD SHGHPRENRT IGATMVRLVR
Microbat                EKAPGKQQRK PQVPGHPMQY MLELYQRSAD VHGHPRENRT IGATMVRLVR
Sloth                   EEVPSKQQRK PRLLGHPLQY MLELYQRSAD LHGHPRENRT IGATMVRMIR
Platypus                GKTP-----K SIFTGQPLQY MLDLYYRSAN QEGLPWENRT LGATSVRLVP
Opossum                 QEVPPKTPQW QPARGRPLQY MLELYRQSAD PQGHPRENRT FRVDAVRLVR
```

```
                                   110       120       130       140       150
                           ....|....| ....|....| ....|....| ....|....| ....|....|
Human                      PLTNVARPHR GTWHIQILGF PLRPNRGLYQ LVRATVVYRH HLQLTRFNLS
Chimpanzee                 PLTNVARPHR GTWHIQILGF PLRPNRGLYQ LVRATVVYRH HLQLTRFNLS
Gorilla                    PLTNVARPHR GTWHIQILGF PLRPNRGLYQ LVRATVVYRH HLQLTRFNLS
Orangutan                  PLTNVARPRR GTWHIQILGF PLRPNRGLYQ LVRATVVYRH HLQLTRRNLS
Macaque                    PLTNVARPRR GTWHIQILGF PLRPNRGIYQ LVRATVVYRH HLQLSRFNLS
Mouse                      PSANTVRPPR GSWHVQTLDF PLASNQVAYE LIRATVVYRH QLHLVNYHLS
Rat                        PSASAMRLLR GPWHIQTLDF PLASNEVAYQ LIRATVVYRH QLHLVHYHLS
Kangaroo rat               PSANVARPLR GSWHIQNLDF PLASNRVAYQ LVRAAVIYRD RLHLARCLLS
Rabbit                     PLANVARPLR GPWHIKTLDF PLRPNRVAYQ LVKATVVYRH QLHLAHFRLS
Sheep                      PLASVARPLR GSWHIQTLDF PLRPNRVAYQ LVRATVVYRH QLHLTHSHLS
Cow                        PLASVARPLR GSWHIQTLDF PLRPNRVAYQ LVRATVVYRH QLHLTHSHLS
Pig                        PLVNGARPLR GPWHIQTLDF PLRPNRVAYQ LVRATVVYRH QLHLAPFHLS
Panda                      PLANVARPLR GPWHIKTLDF PLRPNQVAYQ LVRAIVVYRH QLHLAHFHLS
Dog                        PLANIARPLR GPWHIKTLDF PLRPNRVAYQ LVRAIVVYRH QLYLAPFHLS
Horse                      PLTNVARPLR GPWHIQTLDF PLRSNRVKYQ LVRATVVYRH QLHLSHFNLS
Dolphin                    PLGNVARPLR G-WHKQTLDF PLRPNWVAYQ LVRATVVYRH QLHLAHSHLS
Armadillo                  PLTRIARPFG GPWFIQTLDF PLRTNRVAYQ LVRATVVYHH QLHLAHFHLS
Lesser hedgehog tenrec     PLASVARPLG GSWHVQTLDF PLRPNRVTYK LIKATVVYRH QLHLSHFHFS
Hedgehog                   PLLNIARPLR GPWHMQTLDF PLRSSRVAYQ LVRATVVYRH QLHLNHLHLS
Tarsier                    PLTNAAKPRR GMWHIRTLDF LLRPNRVAYQ LVKATVVYRH QLHLTLFNLS
Microbat                   PLANVARPLR GPRHIQTLDF PWKPNQVAYQ LVRATVVYRH QLHLAGFHLS
Sloth                      PLTRVARPLR GPWHIQTLDF PLRPNQVAYQ LVRATVVYRH QFHLAHFHLT
Platypus                   ASASTGISTR GTWFVQSLEY PMSVVQERQI LVRAAVVYPS VLRKSDTQFL
Opossum                    PAGHRALPPR GPWYVQTLDF PLQPNREGYQ LVRAAVAYRP HLRLSHSHLS

                                   160       170       180       190       200
                           ....|....| ....|....| ....|....| ....|....| ....|....|
Human                      CHVEPWVQKN PTNHFPSSEG DSSKPSL-MS NAWKEMDITQ LVQQRFWNNK
Chimpanzee                 CHVEPWVQKN PTNHFPSSEG DSSKPSL-MS NAWKEMDITQ FVQQRFWNNK
Gorilla                    CHVEPWVQKN PTNHFPSSEG DSSKPSL-MS NAWKEMDITQ LVQQRFWNNK
Orangutan                  CHVEPWVQKN PTNHFSSSEG DSSKPSL-MS NAWKEMDITQ LVQQRFWNNK
Macaque                    CHVEPWVQKS PTKHFPSSEG DLPKSSL-MS NAWKEMDITQ HVQQRFWNNK
Mouse                      CHVETWVPKC RTKHLPSSKS GSSKPSP-MS KAWTEIDITH CIQQKLWNRK
Rat                        CHVEPWVPKC RTKHFPS-KS GSAKPSS-VS KAWREMNITH CIQQKLWNRK
Kangaroo rat               CHVEPWIPKC PVNHFPSSGT GSTKPSL-IP ESWLEMDITH CLQQSLWNRK
Rabbit                     CHVEPWVQKS PTNHLP-PGR GSAKPSL-MS KAWMEMDITQ HIQQRLWNHK
Sheep                      CHVEPWVQKS PTNHFPSSGR GSSKPSL-LP KTWTEMDIME HVGQKLWNHK
Cow                        CHVEPWVQKS PTNHFPSSGR GSSKPSL-LP KAWTEMDIME HVGQKLWNHK
Pig                        CHVEPWIQKS TTSHFPSSGR GSLKPSL-LP QAWTEMDVTQ HVGQKLWNHK
Panda                      CHMEPRVQKS QTNHFPS-GR GSSKPSL-LS KAWTEMDITQ HVQQRLWNPK
Dog                        CHVEPWIQQS LTNHFPSSGR GSSNPSL-MS KAWTEMDITQ HVRQRIWNHK
Horse                      CYVEPWVQKS PTNQFPSSGR VSSKPSL-LS KAWTEMDITQ HIRQRLWNHK
Dolphin                    CHVEPWVQKS PTDHFPSSGS GSSKPSL-LP EAWTEMDITQ HVGQNLWNHK
Armadillo                  CHVEPWVEKR SINHFPSSKR ISSEPSL-LP KVWTEMDITQ HVQQSLWNHR
Lesser hedgehog tenrec     CVVEPWIQKR LTTHFPSSGR GSSDPSL-LN KAWTEMDITK HVQQRLWNHK
Hedgehog                   CHVEPWVQKS QTYSFLPSRR GSSKPSM-LS ETWTEMDITQ HIQQRLWNHK
Tarsier                    CHVEPWVQKS PTNHFFSLGI VSSKPSL-IS KAWTEMDVTQ HVQQRL-NNK
Microbat                   CHVEPWVQKS PTNGFPSSGR GSSKPSL-LS EAWTEMDITQ HVQQRLWNHK
Sloth                      CHVEPWVHKS SINHFPSSNG SSSEPSL-LH KSWTEMDITQ HVWQRLWNHK
Platypus                   CQMKSRIRKN EPHVREPEFE EASSQLY-RP TAWQETDFTA YIRQRLQKAH
Opossum                    CHVEPWAHKS ------SLLL GGGSPGFALP EAWAEMDITN YIQQQFWPQE
```

```
                                  210        220        230        240        250
                         ....|....| ....|....| ....|....| ....|....| ....|....|
Human                    GHRILRLRFM CQQQKDSGGL E-LWHGTSSL DIAFLLLYFN DTHKSIRKAK
Chimpanzee               GHRILRLRFM CQQQKDSGGL E-LWHGTSSL DTAFLLLCFN DTHKSIRKAK
Gorilla                  GHRILRLRFM CQQQKDSGGL E-LWHGTSSL DTAFLLLYFN DTHKSIRKAK
Orangutan                GHRILRLRFM CQQQKDRGGL E-LWHGTSSL DTAFLLLYFN DTHKSIQKAK
Macaque                  GRRILRLRFM CQQQKYSDGL E-LWHGTSSL DIAFLLLYFN DTHKSIQKAK
Mouse                    GRSVLRLRFM CQQQKGNETR EFRWHGMTSL DVAFLLLYFN DTDDRVQ-GK
Rat                      GRRVLRLRFM CQQQKGNETL ELRWHGMTSL DVAFLLLYFN DTDESAQ-AK
Kangaroo rat             GRRFLRFRFM CQQQKGREVL GLQWHGTSSL DIAFLLLYFN DTHKSVQKAK
Rabbit                   GRRVLRLRYM CQQQKGSEVL EFQWPSTSSL DIAFLLLYFN DTHRSIQKAK
Sheep                    GRRVLRLRFV CQQPRGSEVL EFWWHGTSSL DTVFLLLYFN DT-QSVQKTK
Cow                      GRRVLRLRFV CQQPRGSEVR EFWWHGTSSL DTVFLLLYFN DT-QSVQKTK
Pig                      GRRVLRLRFM CQQQNGSEIL EFRGRGISSL DTAFLLLYFN DT-RSVQKAK
Panda                    GCRVLQLRFM CQQQKGSEIL GLQWHGTSSL DTAFLLLYFN DTHKSVQKAK
Dog                      GRRVLQLRLM CQQQKGSEIL ELQWHGTSSL DTAFLLLYFN DTHKSVRKAT
Horse                    GRRVLRLRFV CQQPKDSEVL ELRWHGTSSL DTVFLLLYFN DTHKSGQKTK
Dolphin                  GHRVLRLRFL CQRPRGSEVL EFRWHGTSSL DTAFLLLYFN DT-QSVQKAK
Armadillo                RHRVLRLRFV CQQQKGSEVL EFR-RGTSYF DSAFLLLYFN DTHKSFQKDG
Lesser hedgehog tenrec   GHKILRLLYM CQLQKGSEII EPQWQGTSSM DTAFLLLYLN DTSKSVQKA-
Hedgehog                 GRRILRLNFM CQPQKGSKVL ELRWHGTSSL DTAFLVLYFN DTHKSVRNIK
Tarsier                  RHRILRFRFM CQQQKGSEGL EAQWRGTSAL DIAFLLLYLN DTHKSVQKAK
Microbat                 RRRILRLRFM CQHQKGSEGH ELLWHGTSPL DTAFLLLYFN DTHKSVQKAK
Sloth                    GRRVLRLSFV CQKQKGSEVL ELRWHETSSL DTAFLLLYFN DTHKSFQNSR
Platypus                 GQFSVWLHWK CRQQRARSSS RAPWRGTAAS EAPFLLLYFN DTHQGYQEV-
Opossum                  RRRTLRIQVK CEQQERTN-L GLGWRQALAS DSAFLVLYFN NTFDSMPQV-

                                  260        270
                         ....|....| ....|....| ...
Human                    FLPRGMEEFM ERES-LL--R RTR
Chimpanzee               FLPRGMEEFM ERESLLL--R RTR
Gorilla                  FLPRGMEEFM ERESLLL--R RTR
Orangutan                FLPKGMVEFM ERESLLL--R RTR
Macaque                  LLPRGMEEFM ERESFLL--R RTR
Mouse                    LLARGQEELT DRESSFL-MR SVR
Rat                      LLARGQEELT DRESPFL-MR SVR
Kangaroo rat             PLPRGQQEFV ESESSLF-FR SAR
Rabbit                   APSRGLEEFM ERDSPLL--R RTR
Sheep                    PLPKGLKEFT EKDPSLL-LR RAR
Cow                      PLPKGLKEFT EKDPSLL-LR RAR
Pig                      LLPRGLEEFM ARDPSLL-LR KAR
Panda                    LHPRSLEELM AGDSSLL-LR RAR
Dog                      FHPRVLEGFI EKDSSLL--R RAR
Horse                    LLPRGLEEFM ERDASLL-LR RVR
Dolphin                  LLPRGLEEFT EIDPSLL-LR RAR
Armadillo                FLPR---DFM ESESPIL-SR MAR
Lesser hedgehog tenrec   MLPQSLEEFM ERESPLL-LR KSR
Hedgehog                 VLPRDPKEFM NNDSSLL-LR RTR
Tarsier                  FLPRGLEEFM ERESPLLE-R RTR
Microbat                 LLPGGLEEFM ERDSSLL-LR RAR
Sloth                    HLPRGLEEFM DRESSPL-LR KAR
Platypus                 GPP------- -------LHR RVR
Opossum                  RLQELLTGDP AGADPLLLAR QVR
```
