# Supplementary material for: Positive Selection in Bone Morphogenetic Protein 15 Targets a Natural Mutation Associated with Primary Ovarian Insufficiency in Human
Source: PLoS One. 2013 Oct 16;8(10):e78199. doi: 10.1371/journal.pone.0078199 (PMC3797742; doi:10.1371/journal.pone.0078199)
Supplement: Table S1 — Ensembl protein identification numbers of the 39 members of the TGFbeta family used to draw the phylogenetic tree. (PDF) [file pone.0078199.s002.pdf]

Table S1: Ensembl protein identification numbers of the 39 members of the TGFbeta family used to draw the phylogenetic tree.

Gene	Gene name	Species	Protein ID
**AMH**	anti-Mullerian hormone	Human	ENSP00000221496
		Mouse	ENSMUSP00000043153
		Rat	ENSRNOT00000026220
**BMP15**	bone morphogenetic protein 15	Human	ENSP00000252677
		Mouse	ENSMUSP00000024049
		Rat	ENSRNOP00000003989
**BMP2**	bone morphogenetic protein 2	Human	ENSP00000368104
		Mouse	ENSMUSP00000028836
		Rat	ENSRNOP00000028904
**BMP4**	bone morphogenetic protein 4	Human	ENSP00000394165
		Mouse	ENSMUSP00000073720
		Rat	ENSRNOP00000012957
**BMP5**	bone morphogenetic protein 5	Human	ENSP00000359866
		Mouse	ENSMUSP00000012281
		Rat	ENSRNOP00000014846
**BMP6**	bone morphogenetic protein 6	Human	ENSP00000283147
		Mouse	ENSMUSP00000126999
		Rat	ENSRNOP00000018359
**BMP7**	bone morphogenetic protein 7	Human	ENSP00000379204
		Mouse	ENSMUSP00000009143
		Rat	ENSRNOP00000009656
**GDF5**	growth differentiation factor 5	Human	ENSP00000363489
		Mouse	ENSMUSP00000048079
		Rat	ENSRNOT00000073736
**GDF6**	growth differentiation factor 6	Human	ENSP00000287020
		Mouse	ENSMUSP00000062884
		Rat	ENSRNOP00000010266
**GDF7**	growth differentiation factor 7	Human	ENSP00000272224
		Mouse	ENSMUSP00000038301
		Rat	ENSRNOP00000007928
**GDF9**	growth differentiation factor 9	Human	ENSP00000367942
		Mouse	ENSMUSP00000018382
		Rat	ENSRNOP00000009727
**INHA**	inhibin, alpha	Human	ENSP00000243786
		Mouse	ENSMUSP00000040310
		Rat	ENSRNOP00000027227
**MSTN**	myostatin	Human	ENSP00000260950
		Mouse	ENSMUSP00000027269
		Rat	ENSRNOP00000038159
