# Supplementary material for: Positive Selection in Bone Morphogenetic Protein 15 Targets a Natural Mutation Associated with Primary Ovarian Insufficiency in Human
Source: PLoS One. 2013 Oct 16;8(10):e78199. doi: 10.1371/journal.pone.0078199 (PMC3797742; doi:10.1371/journal.pone.0078199)
Supplement: Table S2 — Ensembl identification numbers (gene, transcript and protein) of the 24 mammalian orthologs of BMP15 used in the branch-site model for positive selection determination. (PDF) [file pone.0078199.s003.pdf]

Table S2: Ensembl identification numbers (gene, transcript and protein, release 70) of the 24 mammalian orthologs of BMP15 used in the branch-site model for positive selection determination. Sheep access numbers are from the NCBI database.

Species	Gene ID	Localization	Transcript ID	Protein ID
**Armadillo**	ENSDNOG00000015377	Scaffold 19158	ENSDNOT00000015376	ENSDNOP00000011918
**Chimpanzee**	ENSPTRG00000021908	Chr. X	ENSPTRT00000040715	ENSPTRP00000037619
**Cow**	ENSBTAG00000045782	Chr. X	ENSBTAT00000064918	ENSBTAP00000056137
**Dog**	ENSCAFG00000016037	Chr. X	ENSCAFT00000025442	ENSCAFP00000023624
**Dolphin**	ENSTTRG00000009353	Scaffold 97013	ENSTTRT00000009348	ENSTTRP00000008864
**Gorilla**	ENSGGOG00000006526	Chr. X	ENSGGOT00000006552	ENSGGOP00000006382
**Hedgehog**	ENSEEUG00000000528	Scaffold 3475	ENSEEUT00000000526	ENSEEUP00000000465
**Horse**	ENSECAG00000023233	Chr. X	ENSECAT00000024874	ENSECAP00000020683
**Human**	ENSG00000130385	Chr. X	ENST00000252677	ENSP00000252677
**Kangaroo rat**	ENSDORG00000013624	Scaffold 6688	ENSDORT00000013622	ENSDORP00000012807
**Lesser hedgehog tenrec**	ENSETEG00000016235	Scaffold 288562	ENSETET00000016233	ENSETEP00000013157
**Macaque**	ENSMMUG00000017358	Chr. X	ENSMMUT00000024410	ENSMMUP00000022846
**Microbat**	ENSMLUG00000003926	Scaffold GL429963	ENSMLUT00000003926	ENSMLUP00000003574
**Mouse**	ENSMUSG00000023279	Chr. X	ENSMUST00000024049	ENSMUSP00000024049
**Opossum**	ENSMODG00000011104	Chr. X	ENSMODT00000014153	ENSMODP00000013900
**Orangutan**	ENSPPYG00000020354	Chr. X	ENSPPYT00000023746	ENSPPYP00000022784
**Panda**	ENSAMEG00000010484	Scaffold GL194739.1	ENSAMET00000011489	ENSAMEP00000011018
**Pig**	ENSSSCG00000012310	Chr. X	ENSSSCT00000013461	ENSSSCP00000013101
**Platypus**	ENSOANG00000013992	UltraContig Ultra315	ENSOANT00000022066	ENSOANP00000022062
**Rabbit**	ENSOCUG00000001641	Scaffold GL018748	ENSOCUT00000001641	ENSOCUP00000020965
**Rat**	ENSRNOG00000002984	Chr. X	ENSRNOT00000003989	ENSRNOP00000003989
**Sheep**	100141303	Chr. X	NM_001114767.1	NP_001108239.1
**Sloth**	ENSCHOG00000008644	Scaffold 53498	ENSCHOT00000008663	ENSCHOP00000007642
**Tarsier**	ENSTSYG00000009502	Scaffold 24322	ENSTSYT00000009499	ENSTSYP00000008716
