# Supplementary material for: Positive Selection in Bone Morphogenetic Protein 15 Targets a Natural Mutation Associated with Primary Ovarian Insufficiency in Human
Source: PLoS One. 2013 Oct 16;8(10):e78199. doi: 10.1371/journal.pone.0078199 (PMC3797742; doi:10.1371/journal.pone.0078199)
Supplement: Table S3 — Branch-site model parameters for positive selection determination. (PDF) [file pone.0078199.s004.pdf]

Table S3: Branch-site model parameters for positive selection determination.

Branches	Model	l [(1)]	Estimates of parameters [(2)]	$2\Delta l$ [(3)]	Positively selected sites (BEB) [(4)]
Opossum	Null	-5910.757048	ρ0 = 0.49, (ρ1= 0.39), ω0 = 0.17, (ω1 = 1)	23.7***	Not allowed
	Alternative	-5898.914814	ρ0 = 0.47, ρ1 = 0.37, (ρ2 = 0.16), ω0 =0.18, (ω1 = 1), ω2 = 100.29		1 site P>90%: 89D; 3 sites P>95%: 55W, 165G, 187P; 1 site P>99%: 73S
Orangutan	Null	-5912.267806	ρ0 = 0.55, (ρ1= 0.45), ω0 = 0.18, (ω1 = 1)	7.63**	Not allowed
	Alternative	-5908.452903	ρ0 = 0.55, ρ1 = 0.42, (ρ2 = 0.17), ω0 = 0.19, (ω1 = 1), ω2 =∞		2 sites P>90%: 25I, 215R ; 1 site P>95% : 146R
Hominidae	Null	-5910.824097	ρ0 = 0.26, (ρ1= 0.19), ω0 = 0.19, (ω1 = 1)	4.74*	Not allowed
	Alternative	-5906.710067	ρ0 = 0.52, ρ1 = 0.39, (ρ2 = 0.09), ω0 = 0.19, (ω1 = 1), ω2=28.45		2 sites P>90%: 146F, 235Y; 1 site P>99%: 189L

(1) Log- likelihood values.

(2) ρ0, ρ1, and ρ2 are the proportions of codons subject to purifying selection, neutral evolution, and positive selection, respectively. ω0, ω1 and ω2 represented dN/dS for each class (purifying selection, neutral evolution and positive selection, respectively).

(3) * significant at $p < 0.05$

** significant at $p < 0.01$

*** significant at $p < 0.001$.

(4) Amino acid numbers referred to opossum sequence (ENSMODP00000013900), orangutan (ENSPPYP00000022784) and human sequence (ENSP00000252677).
