# Supplementary material for: Positive Selection in Bone Morphogenetic Protein 15 Targets a Natural Mutation Associated with Primary Ovarian Insufficiency in Human
Source: PLoS One. 2013 Oct 16;8(10):e78199. doi: 10.1371/journal.pone.0078199 (PMC3797742; doi:10.1371/journal.pone.0078199)
Supplement: Table S4 — Sence and antisense primers used fot human BMP15 mutagenesis. (PDF) [file pone.0078199.s005.pdf]

Table S4: Sense and antisense primers used for human *BMP15* mutagenesis.

Primer	Sequence 5'-3'
F146A forward	CATCTCCAACTAACTCGCGCCAATCTCTCCTGCCAT
F146A reverse	ATGGCAGGAGAGATTGGCGCGAGTTAGTTGGAGATG
L189A forward	GAGATGGATATCACACAAGCTGTTCAGCAAAGGTTCTGG
L189A reverse	CCAGAACCTTTGCTGAACAGCTTGTGTGATATCCATCTC
Y235A forward	GACATTGCCTTCTTGTTACTCGCTTTCAATGATACTCATAAAAGC
Y235A reverse	GCTTTTATGAGTATCATTGAAAGCGAGTAACAAGAAGGCAATGTC
F146S forward	CATCTCCAACTAACTCGCTCCAATCTCTCCTGCCATGTG
F146S reverse	CACATGGCAGGAGAGATTGGAGCGAGTTAGTTGGAGATG
L189H forward	GAGATGGATATCACACAACATGTTCAGCAAAGGTTCTGG
L189H reverse	CCAGAACCTTTGCTGAACATGTTGTGTGATATCCATCTC
Y235C forward	CATTGCCTTCTTGTTACTCTGTTTCAATGATACTCATAAAA
Y235C reverse	TTTTATGAGTATCATTGAAACAGAGTAACAAGAAGGCAATG
